# High levels of fluctuating asymmetry in isolated stickleback populations

**DOI:** 10.1186/1471-2148-12-115

**Published:** 2012-07-12

**Authors:** Nina Trokovic, Gábor Herczeg, Nurul Izza Ab Ghani, Takahito Shikano, Juha Merilä

**Affiliations:** 1 Department of Biosciences, Ecological Genetics Research Unit, University of Helsinki, PO Box 65, FI-00014, Helsinki, Finland

## Abstract

**Background:**

Fluctuating asymmetry (FA), defined as small random deviations from the ideal bilateral symmetry, has been hypothesized to increase in response to both genetic and environmental stress experienced by a population. We compared levels of FA in 12 bilateral meristic traits (*viz.* lateral-line system neuromasts and lateral plates), and heterozygosity in 23 microsatellite loci, among four marine (high piscine predation risk) and four pond (zero piscine predation risk) populations of nine-spined sticklebacks (*Pungitius pungitius*).

**Results:**

Pond sticklebacks had on average three times higher levels of FA than marine fish and this difference was highly significant. Heterozygosity in microsatellite markers was on average two times lower in pond (*H*_E_ ≈ 0.3) than in marine (*H*_E_ ≈ 0.6) populations, and levels of FA and heterozygosity were negatively correlated across populations. However, after controlling for habitat effect on heterozygosity, levels of FA and heterozygosity were uncorrelated.

**Conclusions:**

The fact that levels of FA in traits likely to be important in the context of predator evasion were elevated in ponds compared to marine populations suggests that relaxed selection for homeostasis in ponds lacking predatory fish may be responsible for the observed habitat difference in levels of FA. This inference also aligns with the observation that the levels of genetic variability across the populations did not explain population differences in levels of FA after correcting for habitat effect. Hence, while differences in strength of selection, rather than in the degree of genetic stress could be argued to explain habitat differences in levels of FA, the hypothesis that increased FA in ponds is caused by genetic stress cannot be rejected.

## Background

Three types of asymmetry in bilateral characters have been recognized: directional asymmetry, antisymmetry, and fluctuating asymmetry (FA) [1]. While both directional asymmetry (the same side is consistently larger) and antisymmetry (one of the sides is consistently larger) result from normal development, FA refers to subtle random deviations from perfect symmetry in bilateral traits resulting from developmental perturbations, and is often used as an indicator of stress and/or fitness e.g. [2-7]. The assumption underlying this practise is that FA reflects developmental instability (DI) – an organism’s inability to adjust its development in an ideal symmetric pattern [8]. Several studies have shown that high FA levels are characteristics of individuals with low fitness e.g. [9-12]. The link between FA and various forms of stress has been repeatedly observed: habitat degradation [13], pollution [14], hybridisation [15], inbreeding [16], small population size [17], and marginal distribution [18] have all been associated with increased levels of FA. Therefore, FA has been proposed to be a useful bioindicator of individual quality and/or environmental stress e.g. [2-4]. However, despite these positive results, a number of studies have failed to find the expected relationships between FA and stress or fitness, fuelling a debate about the general applicability of FA as a bioindicator trait in conservation biology for reviews, see [6,19,20]. Numerous analytical and statistical issues, such as the proper control of measurement error in metric traits e.g. [21-23], and the difficulty of reliably estimating DI using single traits [6,7,23,24], might provide at least partial explanation for the conflicting results. These difficulties have also been proposed to account for the recent decrease in popularity of FA studies [6].

While both theory and a number of observations align with the idea that the degree of FA at the individual or population level is indicative of individual quality or degree of stress experienced, relaxed selection against developmental perturbations is also expected to increase FA in given population and/or trait. For instance, several studies have shown that the levels of FA in functionally important bilateral traits is typically less than that in functionally less important traits e.g. [25]. Similarly, it is possible that the degree of canalizing selection against developmental perturbations may differ among different populations. If so, this could provide one explanation for heterogeneity in FA-stress associations in different studies: in two populations experiencing the same incidence of stress induced developmental errors, the one experiencing relaxed selection against FA will express a higher degree of FA on average than a population that is under more stringent normalizing selection. However, to the best of our knowledge, this hypothesis has not been tested to date.

The mechanosensory lateral line system, present in all fishes and aquatic amphibians, has anatomical and functional properties which make it highly suitable and attractive for FA studies. Firstly, the lateral line system consists of numerous sensory receptors (neuromasts) located on the surface of the animal, either superficially (superficial or free neuromasts) or under the skin in fluid-filled canals (canal neuromasts) e.g. [26], which can be counted easily. Meristic traits, such as neuromasts, have been shown to be superior over metric traits in detecting correlations between FA and the environment [20], and can be counted with little error. Secondly, neuromasts are organised in anatomically distinct lines that are distributed bilaterally along the head and trunk, and the existence of multiple traits (i.e. individual lines) provides the possibility to determine the overall level of FA precisely, unlike most single-trait estimations [6]. Thirdly, the lateral line system is functionally very important, and likely to influence individual fitness. This system senses weak water movements and mediates crucial behaviours, including prey detection [27,28], predator avoidance [29], schooling [30], orientation to water currents (rheotaxis) [31], and localization of objects [32-34]. Hence, lateral line asymmetry is likely to reduce fitness, and as such, it is a potential target of natural selection. Further, this effect could be expected to differ among populations living in environments which differ in the demands on the lateral line system.

The goal of the present paper was to compare the degree of FA of marine (high piscine predation risk) and pond (zero piscine predation risk) nine-spined stickleback (*Pungitius pungitius*) populations differing both in the levels of genetic diversity [35] and in the level of expected selection (by piscine predation) for symmetry. Assuming that perfect symmetry in the lateral line system is favoured by natural selection, we hypothesised that either (i) the relaxed selection for symmetry in pond populations under negligible predation, and/or (ii) the reduced genetic variability (= genetic stress) in pond populations, will result in reduced developmental stability in ponds as compared to marine populations. In both cases, one would expect to see higher FA levels in pond than in marine populations. However, because genetic variability varies between populations within the same habitat [35], we also attempted to disentangle the two alternative explanations for increased FA in pond environments.

## Results

**Figure 1:**
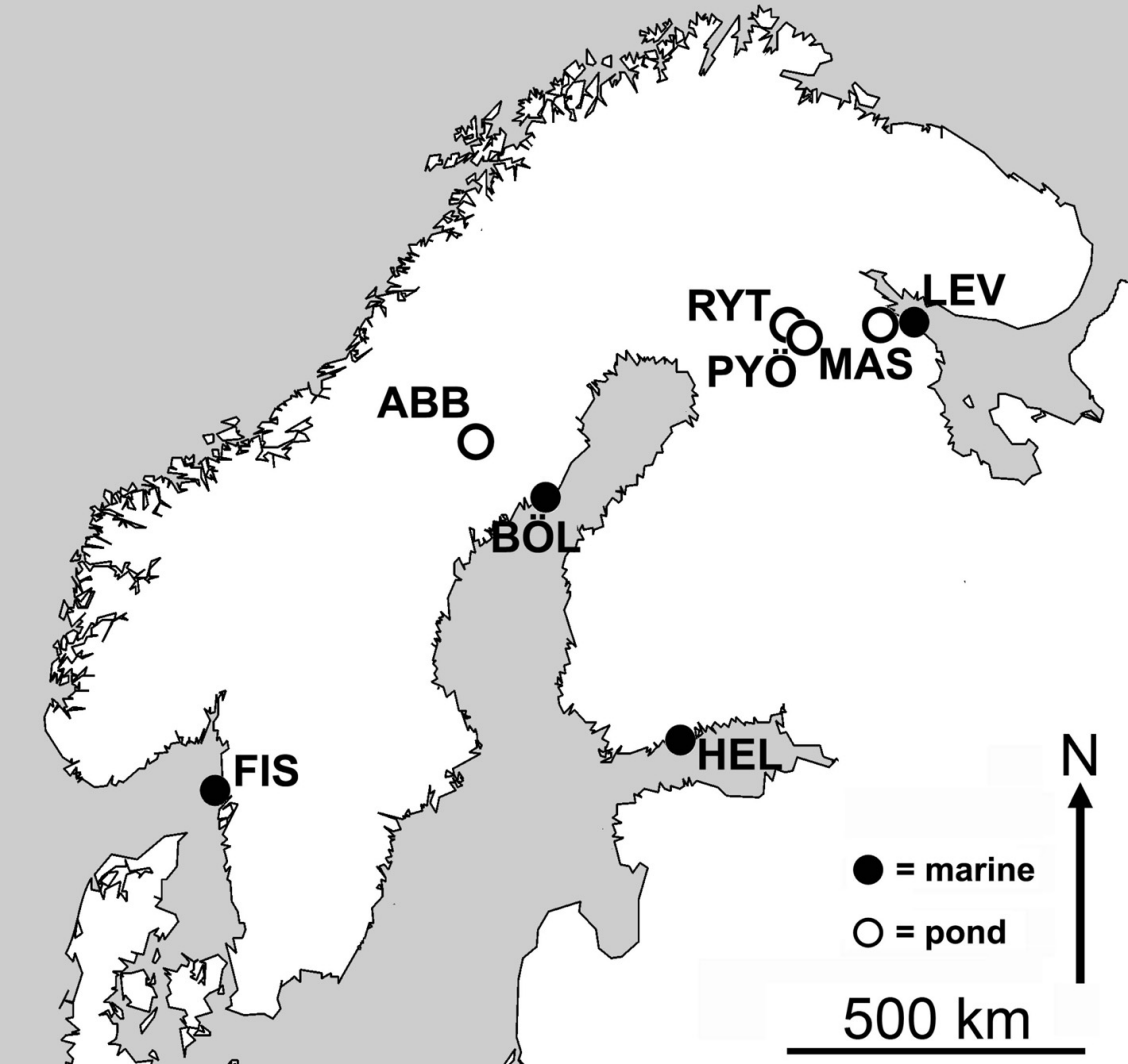
**Average heterozygosity and mean fluctuating asymmetry in eight nine-spined stickleback (* Pungitius pungitius *) populations.** Average (± S.E) heterozygosity was based on screening of variation in 23 microsatellite loci, and mean (± S.E.) fluctuating asymmetry on 12 bilateral traits as estimated by composite standardized relative asymmetry index (see Methods). For population abbreviations, see Figure 2.

The GLMM on heterozygosity revealed a significant habitat effect (*F*_1,6_ = 10.17, *P* = 0.019), but no population effect (*Z* = 1.45, *P* = 0.15). The average (± S.E.) heterozygosity in marine populations (*H*_E_ = 0.58 ± 0.06) was approximately two times higher than in pond populations (*H*_E_ = 0.30 ± 0.06; Figure 1). The GLM revealed a significant population effect (*F*_7,176_ = 14.47, *P* < 0.001) and subsequent post hoc tests revealed no heterogeneity among marine populations (all *P* > 0.22, Figure 1). The Mashinnoje pond population that has only recently become isolated from the White Sea [36]; White Sea Biological Station staff personal communication] did not differ from the marine populations in terms of heterozygosity (all *P* > 0.13, Figure 1). The remaining three ponds (Abbortjärn, Pyöreälampi and Rytilampi) had lower heterozygosity than the marine or Mashinnoje populations (all *P* < 0.01, Figure 1). Pyöreälampi had lower heterozygosity than any of the other populations (all *P* < 0.004, Figure 1). Therefore, while the marine populations had uniformly high heterozygosity, in the ponds heterozygosity varied from being similar to the marine levels (Mashinnoje) to almost zero (Pyöreälampi; Figure 1.).

The GLMM revealed a significant habitat effect (*F*_1,6_ = 35.60, *P* < 0.001) on the composite standardized relative FA-index, irrespective of sex (*F*_1,156_ = 0.27, *P* = 0.60), population (*Z* = 1.05, *P* = 0.30), and heterozygosity (*F*_1,5_ = 2.19, *P* = 0.20). None of the interactions were significant (habitat*sex: *F*_1,143_ = 0.03, *P* = 0.86; habitat*heterozygosity: *F*_1,4_ = 0.10, *P* = 0.76). The pond populations showed almost three times higher levels of asymmetry than the marine populations (Least Squares means ± S.E.; marine = 6.55 ± 1.3; pond = 17.45 ± 1.3; Figure 1). The multivariate GLM supported these results, revealing a strong habitat effect (Wilk’s *λ*_12,141_ = 0.59, *P* < 0.001) on standardized relative asymmetry, irrespective of sex (Wilk’s *λ*_12,140_ = 0.91, *P* = 0.36, habitat*sex: Wilk’s *λ*_12,139_ = 0.96, *P* = 0.91). The population effect was also non-significant (Wilk’s *λ*_72,773_ = 0.54, *P* = 0.07), and the univariate tests showed significant habitat effects for standardized relative asymmetry in all 12 traits (*F*_1,150_ > 5.38, *P* < 0.02). The trends were similar to those revealed by the composite relative asymmetry index: pond populations were generally more asymmetric than marine populations (data not shown).

To explore further the (lack of) heterozygosity effect on FA in the GLMM (see above), we performed simple correlation analyses using the population mean values of the two traits. Using raw values, there was strong negative correlation between mean FA and mean heterozygosity across the eight populations (*r*_*s*_ = - 0.833, *P* = 0.01). However, if the average effect of habitat type is controlled for by performing the correlation using heterozygosity values standardized to a common mean across the habitat types, this correlation disappears (*r*_*s*_ = - 0.071, *P* = 0.35). These results are compatible with the results of the GLMM above, and show that the association between FA and heterozygosity is mainly driven by the association between habitat type and heterozygosity.

## Discussion

The most salient finding of this study was that nine-spined sticklebacks from ponds exhibit significantly and consistently higher levels of FA than their marine conspecifics. Furthermore, while the pond sticklebacks in general had only about half of the genetic variability of marine sticklebacks, the analyses did not support the idea that habitat differences in levels of FA are explainable by differences in heterozygosity once the habitat differences in heterozygosity are controlled for. Hence, the results support the conjecture that high levels of FA in pond populations stem from decreased selection for perfect symmetry, rather than from genetic stress.

Predation is a widely recognized mechanism of natural selection, and some studies have shown that predated individuals express higher levels of FA than surviving individuals e.g. [9,37-39]. Furthermore, decreased FA with age is also suggestive of poorer survival of more asymmetric individuals e.g.[40]. Predation can decrease the population level FA in the prey in at least three ways. First, predation can impose selection for individuals with low FA, resulting in a high degree of developmental canalization and thereby in low FA. However, this implies that there is an additive genetic basis for FA. Heritability of FA is a controversial issue: initial meta-analyses yielded a relatively high average heritability estimate [41], but the subsequent studies have since suggested that the heritability of FA is very low – if not negligible [42,43]. Second, assuming that FA has no, or a weak, genetic basis, and is simply a reflection of the growth environment experienced, predation might simply remove asymmetric individuals from the population. Third, as negligible predation selects for larger body size and higher growth rate [44,45] predation has the potential to affect FA indirectly through altering the growth intensity, where higher growth rate is coupled with higher DI [46]. Regardless of the mechanism, relaxation of predation pressure can be expected to increase the average degree of FA in the population. While we are not aware of any studies that have compared FA levels among populations that differ in selection for perfect symmetry, there are studies which show that less functionally important traits express higher FA than important traits at the individual level e.g. [25]. That said, it is also known that strong directional selection can increase DI in selected traits [47]. However, although the mean number and organisation of lateral-line neuromast differ among marine and pond populations in this species [48], the patterns of differentiation among populations are heterogeneous and directional selection on lateral-line traits is indicated to occur mainly in the marine environment [48]. Likewise, the lateral plate number – one of the traits analysed in study – is shown to be reduced in pond as compared marine populations presumably as response relaxed piscine predation in pond environments [49]. Hence, it seems unlikely that the increased DI in pond populations’ lateral-line traits and lateral plate numbers would results from directional selection.

Perhaps the most marked difference between pond and marine nine-spined stickleback populations is the predation risk; marine sticklebacks are sympatric to a large number of predatory fish species, while ponds lack predatory fish and the nine-spined stickleback is often the only fish species present in ponds [49,50]. Previous studies have demonstrated marked behavioral and morphological differences in nine-spined sticklebacks in relation to predation risk [44,49,50], including a recent study demonstrating habitat and population specific differences in the lateral line system [48]. The mechanosensory lateral line system of fish and aquatic amphibians responds to weak water movements and is involved in avoidance of predators [29,49,51] and in schooling [30], which is an important antipredator behaviour [52]. Hence the negligible predation in ponds might have resulted in relaxed selection for perfect symmetry in the lateral line system and consequently, in the high levels of FA observed in this habitat. While this is, to the best of our knowledge, the first study to suggest this effect, we admit that the exact functions of the different lateral-line traits in this species are as yet unknown [26,48]. Hence, further functional studies about how the information from the lateral-line is used in different contexts are needed. However, the fact that levels of FA in lateral plate numbers – a trait associated with variation in piscine predation [49] – showed exactly the same patterns of FA as lateral-line traits supports the importance of predation in dictating the observed patterns.

Inbreeding (mating among relatives) can increase homozygosity and result in inbreeding depression, which can manifest itself in reduced survival and fertility e.g. [52,53]. Increased FA levels have been linked to reduced heterozygosity both in the field e.g. [16,54,55], and in controlled laboratory experiments with induced inbreeding e.g. [56]. It has been shown that pond nine-spined stickleback populations have lower genetic variability than marine populations [35], and one explanation for the higher level of FA in pond sticklebacks could be that reduced genetic variability in pond populations has resulted in increased DI, and consequently increased FA. Based on the populations used in this study, the heterozygosity of pond populations was on average half that of marine populations, with heterozygosity being highly variable among pond populations, but similar among marine populations. However, formal tests – accounting for the on average lower heterozygosity in pond populations – failed to find association between heterozygosity and FA across the populations. This finding is not completely surprising, as some other studies also found that heterozygosity had a weak, or no effect on FA [57-60]. However, given the fact heterozygosity and habitat type are tightly associated in our study, their independent effects on FA cannot be fully disentangled.

Obviously, there are other factors that potentially affect FA that we could not directly address here. For instance, there might be environmental stressors (e.g. water quality, temperature, oxygen levels, pH, etc.) that may differ among marine and pond populations, and cause higher levels of FA in ponds. At the moment, too little is known about the variation in relevant environmental parameters and their potential impact on FA in pond vs. marine habitats to form informed arguments about their significance, but it is worth noting that there is no *a priori* reason to suggest that pond fish would experience more stressful environmental than the marine fish. In fact, pond fish live longer, grow faster and attain larger sizes than marine fish both in laboratory and the wild [44,45]. Nevertheless, more environmental data coupled with experiments conducted under common garden settings would be needed to study possible environmental determinants of high FA in pond fish. However, irrespectively of the causes, the fact remains that the levels of FA in pond populations are markedly elevated as compared to marine populations.

## Conclusions

In conclusion, the results demonstrate that there is a three-fold difference in levels of FA between pond and marine nine-spined stickleback populations (pond > marine). While there is also a two-fold difference in heterozygosity (pond < marine), the loss of genetic variation did not explain the divergence in levels of FA once the habitat differences in heterozygosity were controlled for. We hypothesize that the negligible predation in pond populations (contrasted to the high predation in marine environments) is responsible for the increased FA in ponds.

## Methods

### Study populations and data collection

**Figure 2:**
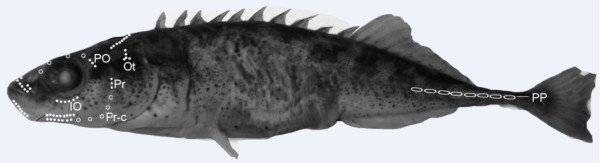
**Map of Fennoscandia showing the localities of the nine-spined stickleback (* Pungitius pungitius *) populations used in our study.** FIS = Fiskebäckskil, Atlantic Ocean, Sweden; BÖL = Bölesviken, Baltic Sea, Sweden; HEL = Helsinki, Baltic Sea, Finland; LEV = Levin Navolok Bay, White Sea, Russia; ABB = Abbortjärn, Sweden; RYT = Rytilampi, Finland; PYÖ = Pyöreälampi, Finland; MAS = Mashinnoje, Russia.

The nine-spined stickleback is a small-bodied teleost fish with a circumpolar distribution, which occurs in various habitats that differ with respect to both biotic and abiotic stress [61]. Adult nine-spined sticklebacks were collected using minnow traps and seine nets during the breeding seasons (May- June) between 2007 and 2009. The fish were collected from four ponds and four marine populations from geographically distinct locations in Fennoscandia (Figure 2). Coastal marine environments represent ecologically complex habitats with diverse fish communities, and a large number of potential predator fish species that can predate on every age and size group of nine-spined sticklebacks, while ponds, which are extremely small (surface area < 5 ha) and completely isolated, lack predatory fish [44]. The only sympatric fish species in our study ponds was the three-spined stickleback (*Gasterosteus aculeatus*) in Mashinnoje, and recently introduced small-bodied whitefish (*Coregonus lavaretus*) in Pyöreälampi, both of which are potential competitors, but not predators, of nine-spined sticklebacks. We note that apart from predation by fish, sticklebacks are also predated by aquatic insects, birds and conspecifics. However, according to our observations, bird predators in our study ponds are extremely scarce if not absent, whereas they are numerous at the marine sites [Gábor Herczeg & Juha Merilä personal observations]. Predation by aquatic insects and adult conspecifics might be relevant in all populations to a certain degree; we have no quantitative data on these effects. However, several lines of independent evidence suggest that the predation regime in pond and marine populations differ drastically. Namely, pond sticklebacks have reduced or absent defensive body armour, live almost two times longer on average, and behave bolder when compared to marine fish [44,49,50]. Hence, the difference in predation risk by piscine predators appears to be defining feature differentiating marine and pond populations in focus of this study.

**Figure 3:**
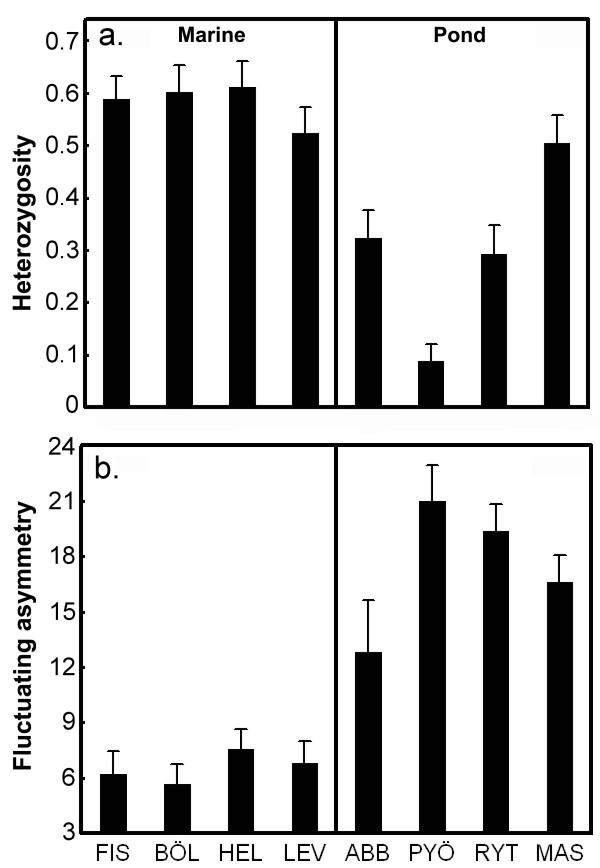
**Schematic presentation of the location of the lateral line and lateral plate traits.** Canal neuromast pores are shown as open circles, superficial neuromasts as dots. Shown are also posterior plates (PP), and lateral line structures: dorsal head (DH), infraorbital (IO), mandibular (M), mandibular preopercular lower (MPrL), mandibular preopercular upper (MPrU), nasal (N), otic (Ot), postorbital (PO), preopercular (Pr), preopercular canal (Pr-c), supraorbital canal (SO-c).

Collected fish were over-anesthetized with MS 222 (tricaine methanesulfonate) at the site of capture, and stored in 96 % ethanol. Samples were later fixed in 4 % formalin. A standard bone-staining procedure was used for the visualization of neuromasts. In short, the fish samples were briefly dehydrated in 70 % ethanol, and placed in 1 g/l alizarin red; 0.5 % KOH for 3 days. Fish were destained in 1 % KOH for 4 days and transferred to alcohol. Posterior lateral plates and neuromasts from 11 lateral lines (Figure 3) were counted under a dissecting microscope (Wild M5A; Wild, Heerbrugg, Switzerland). The use of meristic characters instead of metric ones is beneficial, because the former can be recorded (in theory) with perfect accuracy, while the latter can not. We used 20 individuals from every population. We aimed to measure 10 males and 10 females per population, but in two marine populations (Helsinki, Levin Navolok Bay; see Figure 2) we only obtained females, so we used 20 females for these populations. Twenty individuals were counted twice for every trait. The repeatability (*R*) of the left side – right side values were high (mean *R* = 0.97, median *R* = 1, minimum-maximum: 0.75 – 1, *P* < 0.001 in all tests).

Since this was a non-experimental study involving only collection of animals, no ethical permissions were required. Nine-spined stickleback is not protected in any of the sampled countries, hence, sampling permits from local conservation authorities were not needed in general. However, one of the sampled areas (Oulanka region in Finland) included a protected area, and for sampling in the water bodies in that particular area, we requested and received sampling permit from the responsible conservation authority, Metsähallitus (2007, permit given for GH). To import the samples to Finland, we requested and received permits from the Finnish Food Safety Authority (EVIRA, # 2736/425/2007, 3342/425/2007, 577/0527/2009).

### Genetic analyses

DNA was extracted from fin clips using a phenol-chloroform [62] or a silica-fine based purification method [63], following proteinase K digestion. The following 23 microsatellite loci were genotyped: Gac1125PBBE, Gac4174PBBE, Gac7033PBBE, GAest3, GAest7, GAest14, GAest35, GAest50, GAest66, GAest82, Stn49, Stn71, Stn89, Stn96, Stn100, Stn127, Stn130, Stn163, Stn173, Stn196, Stn198, Stn223 and Stn253 [64-68]. The forward primers were labelled with fluorescent dyes (FAM, HEX or TET) for visualization of the PCR products, and the 5’-end of the reverse primers was modified with an additional GTTT-tail to enhance the 3’-adenylation [69]. The loci were arranged in multiplex PCR panels with non-overlapping size ranges, and all amplifications were carried out using the Qiagen Multiplex PCR Kit (Qiagen) containing 1× Qiagen Multiplex PCR Master Mix, 0.5× Q-Solution, 2 ρmol of each primer, 10–20 ng of template DNA and MQ water for a final reaction volume of 10 μl. PCRs were performed using the following cycle: an initial denaturation step at 95 °C for 15 min, followed by 30 s at 94 °C, 90 s at 53 °C and 60 s at 72 °C for 30 cycles, with a final extension at 60 °C for 5 min. PCR products were separated using a MegaBACE 1000 automated sequencer (Amersham Biosciences) and their sizes were determined using ET-ROX 400 or 550 size standard (Amersham Biosciences). Alleles were scored using Fragment Profiler 1.2 (Amersham Biosciences), with visual inspection and manual correction. Some (11 loci for seven populations) of the microsatellite genotypic data used in here are a subset of data from Shikano *et al.*[35]. Genetic diversities (*H*_E_) were estimated using FSTAT 2.9.3 [70]. To verify that the used genetic markers behave as neutral, we also conducted an outlier test using the program LOSITAN [71]. The results suggest that none of the used microsatellite loci have been subject to recent directional selection. Although three loci (GAest14, GAest50 and GAest66) were indicated to be under balancing selection, we retained them in the analyses because of the methodological problems associated with the identification of balancing selection in outlier tests [72]. We also note that our estimates of heterozygosity in 23 microsatellite loci are likely to reflect true genome-wide heterozygosity [c.f. [73] in these populations: correlation between heterozygosity in the 12 of the microsatellite loci also used here and 15 318 SNP loci across eight nine-spined stickleback populations has been found to be very high (*r* = 0.97; Johnston S., et al, submitted]). Likewise, mean heterozygosity in the 23 markers used in this study is strongly correlated with mean heterozygosity in abovementioned SNPs across five populations common with this and the SNP study (*r* = 0.92). Furthermore, a subset (n = 5) of populations used in this study have also been genotyped for 112 microsatellite loci (T. Shikano, unpublished data) and the correlation between estimates based on 112 *vs*. 23 markers is high (*r* = 0.96). Hence, the population specific heterozygosity estimates are likely to be good estimators of genome-wide heterozygosity.

### Statistical analyses

To compare the levels of heterozygosity between the populations, we used locus- and population-specific heterozygosity estimates. First, we used a General Linear Mixed Model (GLMM) to test for habitat effects. Here, heterozygosity estimated for the different loci was the dependent variable, habitat type (marine *vs.* pond) the fixed factor, and population nested within habitat a random factor. Second, to have population-based pairwise comparisons, we ran a General Linear Model (GLM) with heterozygosity as the dependent variable and population as a fixed factor, followed by pairwise Fisher LSD post hoc tests.

To ensure that the analysed asymmetry is FA, one has to exclude the possibility that directional asymmetry and antisymmetry [see e.g. 1] are responsible for the patterns. To test for the presence of directional asymmetry, we compared the left side–right side values by trait and population to the hypothetical zero value using one-sample t-tests. Out of the 96 tests, only four cases were significant (data not shown). Considering that the four significant cases represented (i) different traits in different populations, (ii) only 4.2 % of all tests, and (iii) there were 12 non-independent tests for every population, we can be confident that the measured asymmetry was not directional asymmetry. The presence of antisymmetry was excluded after visual inspection of the distributions of the left – right side values for every trait and every population separately.

We performed two analyses to look at FA with multiple traits. For the first analysis, we calculated a composite FA-index for each individual based on all traits. This index was modified after Leung et al.’s [7] recommended Composite Fluctuating Asymmetry index 2 (CFA 2). We initially calculated relative asymmetry to take the mean number of counts for a given trait into account [74,75]:

(1) $$RA_{i,j}=\frac{\left|L_{i,j}-R_{i,j}\right|}{\frac{L_{i,j}+R_{i,j}}{2}}$$

where the relative asymmetry (*RA*) for individual *i*’s trait *j* is given based on the left (*L*) and right hand (*R*) counts. This step is important because there is a big developmental difference between hypothetical cases with one vs. two, or 101 vs. 102, counts in the two sides. Using this *RA* instead of the absolute difference between the sides, we then followed Leung et al.’s [7] CFA 2 procedure by first dividing individual *RA* values with the mean *RA* found in the given trait across all individuals to control for possible differences in the relationship between FA and DI in different traits:

(2) $$SRA_{i,j}=\frac{RA_{i,j}}{\bar{RA_{j}}}$$

and then summarized these standardized relative asymmetry values (*SRA*) across all traits for every individual:

(3) $$CSRA_{i}=\sum SRA_{i}$$

Composite standardized relative asymmetry (*CSRA*) describes the individual level asymmetry considering every trait with equal weight. *CSRA* was then analysed with a GLMM with habitat (marine *vs.* pond) and sex as fixed factors, heterozygosity as a covariate, and population nested within habitat as a random factor.

For the second analysis, we ran a multivariate GLM with the trait-based *SRA* (2) values as dependent variables, and with habitat, sex and population nested within habitat as fixed factors. Upon significant multivariate effects, we evaluated the subsequent univariate tests. Note that no random factor can be fitted in multivariate models, and hence, we had to enter population nested within habitat as a fixed factor. For this reason we could not test for the effects of (population level) heterozygosity and habitat in the same model, and thus had to drop heterozygosity from this second approach. However, as simulations have shown that the multivariate GLM (MANOVA) approach is inferior compared to the use of standardized, summed FA values across traits similar to our *CSRA*[7], we only used the multivariate GLM as a means to see whether the habitat-dependent patterns revealed by the GLMM on *CSRA* (see Results, heterozygosity had no effect) can be found on a trait-by-trait basis too.

In all models, we included the habitat × sex interaction, and in the GLMM, the habitat × heterozygosity interaction was also included. To avoid misinterpretation of the data, the non-significant terms from the models were removed e.g. [76] using backward stepwise models selection based on the *P* < 0.05 criterion see e.g. [77]. This method for model selection is conservative in comparison with those based on Akaike’s or Bayesian information criteria (AIC or BIC), and differs very little from the others in its predictive ability [78]. We mentioned earlier that two of the eight populations lacked males, and so the sex effects should be interpreted accordingly. All analyses were done using SPSS 15.0 for Windows (SPSS Inc. Chicago, Illinois).

## Competing interests

The authors declare no competing interests.

## Authors’ contributions

NT conducted the lateral line counting and conceived and designed the study together with JM and GH. GH and JM collected the samples. TS and NIAG carried out the genetic analyses. GH, NT and JM conducted the statistical analyses. NT and JM finalized the manuscript draft with help from other authors. All authors read and approved the final manuscript.
